# Acetylcysteine for prevention of contrast-induced nephropathy after intravascular angiography: A systematic review and meta-analysis

**DOI:** 10.1186/1741-7015-2-38

**Published:** 2004-10-22

**Authors:** Sean M Bagshaw, William A Ghali

**Affiliations:** 1 Department of Critical Care Medicine, Calgary Health Region, Calgary, Canada; 2 Department of Medicine, University of Calgary, Calgary, Canada; 3 Department of Community Health Sciences, University of Calgary, Calgary, Canada; 4 Centre for Health and Policy Studies, University of Calgary, Calgary, Canada

## Abstract

**Background:**

Contrast-induced nephropathy is an important cause of acute renal failure. We assess the efficacy of acetylcysteine for prevention of contrast-induced nephropathy among patients undergoing intravascular angiography.

**Methods:**

We conducted a systematic review and meta-analysis of randomized controlled trials comparing prophylactic acetylcysteine plus hydration versus hydration alone in patients undergoing intravascular angiography. Studies were identified by searching MEDLINE, EMBASE, and CENTRAL databases. Our main outcome measures were the risk of contrast-induced nephropathy and the difference in serum creatinine between acetylcysteine and control groups at 48 h.

**Results:**

Fourteen studies involving 1261 patients were identified and included for analysis, and findings were heterogeneous across studies. Acetylcysteine was associated with a significantly reduced incidence of contrast-induced nephropathy in five studies, and no difference in the other nine (with a trend toward a *higher *incidence in six of the latter studies). The pooled odds ratio for contrast-induced nephropathy with acetylcysteine relative to control was 0.54 (95% CI, 0.32–0.91, p = 0.02) and the pooled estimate of difference in 48-h serum creatinine for acetylcysteine relative to control was -7.2 μmol/L (95% CI -19.7 to 5.3, p = 0.26). These pooled values need to be interpreted cautiously because of the heterogeneity across studies, and due to evidence of publication bias. Meta-regression suggested that the heterogeneity might be partially explained by whether the angiography was performed electively or as emergency.

**Conclusion:**

These findings indicate that published studies of acetylcysteine for prevention of contrast-induced nephropathy yield inconsistent results. The efficacy of acetylcysteine will remain uncertain unless a large well-designed multi-center trial is performed.

## Background

Contrast-induced nephropathy is a leading cause for acquired acute reductions in kidney function [1,2]. Despite advances in supportive therapy, the incidence of contrast-induced nephropathy may continue to increase significantly with the broader utilization of radiocontrast media for diagnostic and interventional procedures.[3] Furthermore, contrast-induced nephropathy is associated with a greater risk of in-hospital morbidity, mortality, prolonged hospitalization, increased health care costs and potentially irreversible reduction in kidney function [4-8].

The pathophysiology of contrast-induced nephropathy remains incompletely understood. However, current evidence suggests that contrast media induce prolonged vasoconstriction and medullary ischemia coupled with generation of free radicals and oxidative injury to tubular cells [9-11].

Acetylcysteine, a thiol-containing anti-oxidant, has been hypothesized to prevent contrast-induced nephropathy. The potential benefit of acetylcysteine is believed to be mediated by its properties as a scavenger of free-radical species and by increasing the synthesis of nitric oxide, a potent vasodilator, in response to ischemic or other toxic injury in the kidney [12]. Given the recent publication of a series of randomized controlled trials assessing the efficacy of acetylcysteine in preventing the decline in kidney function following contrast exposure associated with intravascular angiography, we sought to conduct a systematic review and meta-analysis of these trials. The specific objectives of our meta-analysis were to assess the effect of acetycysteine on 1) the dichotomous endpoint of contrast-induced nephropathy (yes/no) and 2) serum creatinine levels following the administration of contrast media. We also conduct a meta-regression analysis to determine whether particular clinical or study quality factors influence the apparent effect of acetylcysteine on risk of contrast-induced nephropathy.

## Methods

### Search strategy

We identified published randomized controlled trials of acetylcysteine for prevention of contrast-induced nephropathy during intravascular angiography using both electronic and manual search strategies. We supplemented this by scanning the reference lists of all identified articles, reviewing selected conference proceedings, and by contacting experts in the field. All languages and types of publications were considered eligible. The comprehensive literature search was initially performed in April 2003 and updated in June 2004 to identify any potential new studies that may have appeared.

MEDLINE (1966 through April, 2003), EMBASE (1980 through April, 2003) and CENTRAL (Cochrane Controlled Clinical Trials Register 1996 through April, 2003) databases were searched via OVID using an approach recommended for systematic reviews of randomized trials [13]. PubMed was also searched [14]. We derived three comprehensive search themes that were then combined using the Boolean operator 'and'. The first theme used a recommended highly sensitive randomized controlled trial filter and systematic review filter method [15]. The second theme, contrast-induced nephropathy, was created by using the Boolean search term 'or' to search for the following terms appearing as both exploded medical subject headings (MeSH) or text words: 'contrast media' or 'radiocontrast' or 'kidney failure' or 'acute renal failure' or ' chronic renal failure' or 'contrast nephropathy' or 'dialysis'. The third theme, acetylcysteine, was created by a search using an exploded MeSH heading and textword search for: 'N-acetylcysteine' or 'NAC' or 'acetylcysteine' or 'Mucomyst'.

### Study selection criteria

Two individuals (SMB and WAG) independently evaluated identified articles for eligibility on the basis of four inclusion criteria: 1) study design (randomized controlled trials), 2) target population (patients undergoing intravascular angiography), 3) intervention (trials of acetylcysteine plus hydration versus control) and 4) outcome (trials with explicit definition of contrast-induced nephropathy).

### Data extraction

Two reviewers (SMB and WAG) independently extracted data from all primary studies fulfilling eligibility criteria. Any discrepancies in extracted data were resolved by consensus. Data extracted included identifying information, focus of the study, details of study protocol and demographic data. The primary outcome measures were the incidence of contrast-induced nephropathy and change in serum creatinine. The secondary outcome measure was requirement for renal replacement therapy. Authors of the studies were contacted for additional information when applicable.

### Assessment of methodological quality

Two reviewers (SMB and WAG) independently assessed methodological quality of individual studies. Any disagreements were resolved by consensus. Items used to assess study quality were methods of randomization, any blinding, use of a placebo, reporting of losses to follow-up or missing outcome assessments, and evidence of important baseline differences between the groups [16-18]. An overall quality score was determined for each study as described by Jadad *et al *[16].

### Prior hypotheses regarding sources of heterogeneity

The presence of heterogeneity can compromise the interpretation and validity of meta-analyses and can result from significant differences in methodology, study populations, interventions, outcomes, or chance [19]. A priori consideration of potential factors contributing to heterogeneity for acetylcysteine in prevention of contrast-induced nephropathy included baseline serum creatinine levels, volume of contrast media, volume of hydration, age, diabetes mellitus, elective or emergency procedure, and a number of trial methodology factors.

### Statistical methods

Data from all of the selected randomized controlled trials were combined to estimate the pooled odds ratio (OR) with 95% confidence intervals (CIs) using a random-effects model as described by Der Simonian and Laird [20,21]. The presence of heterogeneity across trials was evaluated using a chi-square test for homogeneity [22]. Meta-regression was performed to analyze for potential clinical and study quality factors that may influence treatment effects. We tested for potential publication bias using both a Begg's test for asymmetry and an Egger's test [23,24]. All statistical analyses were performed with Stata version 8.0 (StataCorp, College Station, TX).

## Results

### Identification of studies

**Figure 1:**
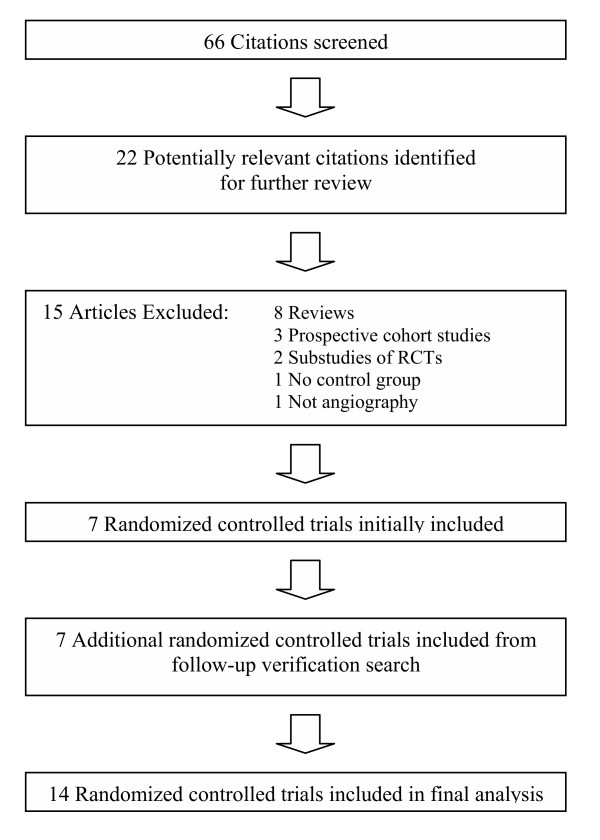
Flow diagram of study selection process.

A total of 66 unique citations were identified by our initial search strategy (Figure 1). After the initial screen, 22 citations warranted further review. Among these, 15 citations were excluded: 8 were clinical reviews, 3 were prospective cohort studies, 2 were substudies of previously published randomized controlled trials, one did not include a control group, and one did not involve intravascular angiography. Therefore, we had identified 7 studies for inclusion. A repeat search of the literature conducted in June 2004 yielded seven additional eligible studies. Overall, 14 studies thus fulfilled our inclusion criteria [25-38]. All of these citations were identified by the electronic search strategy and are published in peer-reviewed journals [39].

### Study characteristics

All the randomized controlled trials were published in the years 2002 through 2004. Tables 1 and 2 present the characteristics of the 14 randomized controlled trials. A total of 1261 patients were studied in these 14 randomized controlled trials, among whom 631 received acetylcysteine and 630 were in control groups. There were 563 (44.6%) patients with diabetes mellitus, of whom 284 were assigned to receive acetylcysteine and 279 were assigned to a control group. The dosing and schedule of administration of acetylcysteine was variable across studies; however, in the majority of studies, acetylcysteine was initiated 12–24 h prior to angiography. In two trials, large doses of acetylcysteine were administered immediately prior to (within 1 h) and shortly following (within 3–4 h) angiography [26,29]. All patients were administered a hydration protocol around their procedure and all received low or iso-osmolar non-ionic contrast media.

The definition of contrast-induced nephropathy was variable across studies. Four studies defined contrast-induced nephropathy as a > 44.2 μmol/L increase in serum creatinine from baseline [25,29,32,37], four used a > 25% increase in serum creatinine from baseline [26,30,33,35], four used either a > 44.2 μmol/L or a > 25% increase in serum creatinine from baseline [28,31,34,36], one used either a > 44.2 μmol/L or a > 33% increase in serum creatinine from baseline[38] and one study combined either a > 25% increase in serum creatinine from baseline or dialysis [27]. Generally, the time for ascertaining contrast-induced nephropathy for all studies was 48 h after the exposure to contrast media, with the exception of four studies, where presence or absence of contrast-induced nephropathy was determined at 24, 72 and 96 h [26,30,34,35].

### Meta-analysis of incidence of contrast-induced nephropathy

**Figure 2:**
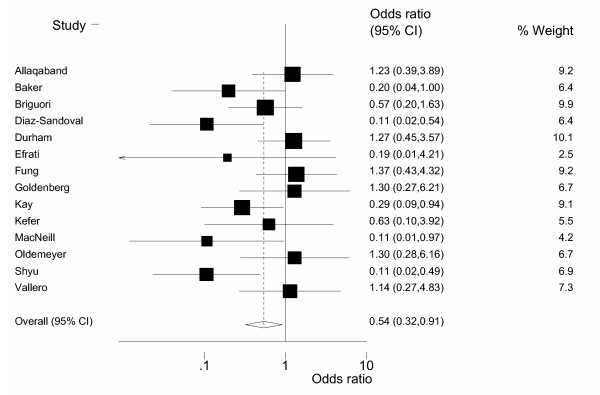
Forest plot of odds ratios for development of contrast-induced nephropathy from 14 trials.

The reported incidence of contrast-induced nephropathy was variable across studies. Table 3 and Figure 2 present information on the incidence of contrast-induced nephropathy for all studies. Five studies provided evidence of a risk reduction for development of contrast-induced nephropathy with acetylcysteine [26,28,33,35,37], whereas nine studies reported no evidence of benefit [25,27,29-32,34,36,38]. Furthermore, six of the latter studies yielded an odds ratio > 1.0, suggesting a trend towards an increased risk of contrast-induced nephropathy [25,29,31,32,36,38]. The overall pooled odds ratio for development of contrast-induced nephropathy using a random-effects model was 0.54 (95% CI, 0.32–0.91, p = 0.022), suggesting a significant reduction in CIN with acetylcysteine (Figure 2). However, this pooled odds ratio should be interpreted with caution because the analysis comparing the occurrence of contrast-induced nephropathy across all studies revealed significant heterogeneity (chi-square = 23.96, p = 0.032). In total, six patients required dialysis, among whom two received acetylcysteine and two were in control groups. Group assignment was not reported for the other two patients who required dialysis.

### Meta-analysis of change in serum creatinine with acetylcysteine

**Figure 3:**
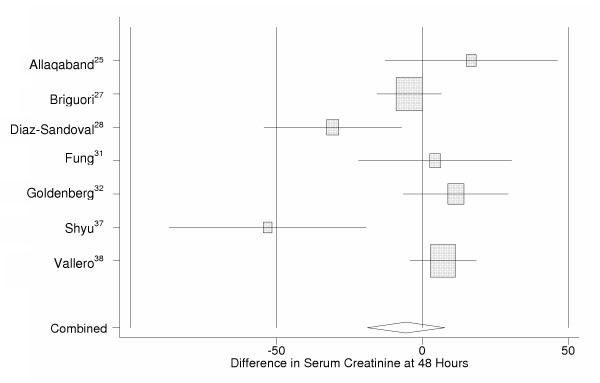
Forest plot of differences in serum creatinine between acetylcysteine and control at 48 h after contrast media administration from eight trials.

Table 3 shows a summary of the changes in serum creatinine across studies. The pooled estimate (using a random effects model) for the difference in 48 h serum creatinine between the acetylcysteine and control groups was -7.2 μmol/L (95% CI -19.7 to 5.3, p = 0.26) based on data available from eight studies [25,27,28,31,32,35,37,38]. This suggests no significant absolute change in serum creatinine with the administration of acetylcysteine (Figure 3). Again, this pooled estimate requires cautious interpretation owing to the availability of data from only eight studies and to the presence of significant heterogeneity across studies (Q = 50.9, p < 0.0005). The change in serum creatinine at 96 h was assessed in two studies as a primary outcome [26,30]. The pooled estimate for the difference in 96-h serum creatinine for these two studies was similarly non-significant [-1.8 μmol/L (95% CI -8.9 to 5.2, p = 0.61)].

### Meta-regression

Meta-regression was performed to assess a number of clinical and study quality factors that may have led to heterogeneity across studies. Interestingly, these analyses suggest that the heterogeneity may be partially explained by whether the angiography procedures were performed electively or as emergency, because studies where all enrolled patients were undergoing elective procedures had significantly lower odds ratios than did studies where emergency cases were included (coefficient for "elective-only" studies, -0.6, 95% CI, -1.24 to 0.03, p = 0.06).

Other meta-regression analyses demonstrated that the heterogeneity could not be accounted for by differences in patient age (coefficient -0.04, 95% CI, -0.2 to 0.1, p = 0.6), baseline serum creatinine (coefficient -0.001, 95% CI, -0.01 to 0.01, p = 0.9), volume of contrast media (- 0.006, 95% CI, -0.02 to 0.07, p = 0.4) or diabetes mellitus (coefficient -0.01, 95% CI, -0.03 to 0.02, p = 0.6). Likewise, heterogeneity was not accounted for by differences in study quality including use of blinding (coefficient -0.6, 95% CI, -1.7 to 0.5, p = 0.3), concealment of randomization (coefficient -0.8, 95% CI, -3.8 to 2.1, p = 0.6), use of placebo (coefficient -0.6, 95% CI, -1.7 to 0.5, p = 0.30, consecutive patient enrollment (coefficient 0.5, 95% CI, -1.5 to 2.4, p = 0.6) or overall Jadad score (coefficient 0.05, 95% CI, -0.4 to 0.5, p = 0.8).

**Figure 4:**
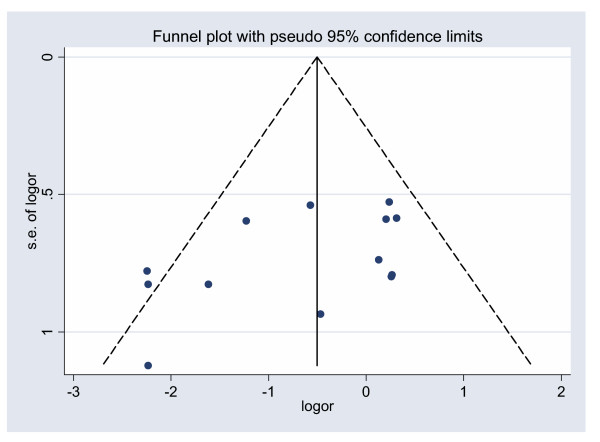
Evidence of publication bias by Funnel plot. Funnel plot asymmetry is demonstrated by evidence of a cluster of small studies with low-protective odds ratio and the paucity of small negative studies in the lower right of the funnel plot.

There was some evidence to suggest possible publication bias according to Begg's test (p = 0.03, with continuity correction) and a trend with Egger's test (coefficient -3.03, 95% CI, -6.71 to 0.65, p = 0.09). Figure 4 demonstrates this graphically, as there is asymmetry in the funnel plot with a predominance of studies with large standard errors (i.e., usually small studies) showing benefit associated with acetylcysteine and a paucity of small negative studies.

## Discussion

Our meta-analysis of 14 peer-reviewed studies of patients undergoing intravascular angiography may lead some to conclude that the administration of acetylcysteine causes a reduced incidence of contrast-induced nephropathy. However, such a conclusion may be premature based on data published to date because our systematic review reveals considerable heterogeneity of findings across trials. Furthermore, our meta-analysis of post-treatment creatinine values does not reveal any truly meaningful difference in serum creatinine levels at 48 h between the acetylcysteine and control groups. Finally, insufficient data are available to allow inferences to be drawn about the efficacy of acetylcysteine on clinically meaningful endpoints such as dialysis, length of hospitalization or mortality.

This meta-analysis has several features that distinguish it from a similar meta-analysis by Birck *et al *that recently received considerable attention, and that rather firmly concluded that acetylcysteine is beneficial [40]. First, though our meta-analysis yielded a similar overall reduction in the incidence of contrast-induced nephropathy, we have included seven additional studies. Second, we have focused primarily on patients undergoing intravascular angiography. Third, we have used the pooled odds ratio across studies as a summary statistic because of its theoretical advantage to the use of relative risks in meta-analysis [21]. Fourth, we have included an analysis of differences in serum creatinine to complement the dichotomous endpoint of contrast-induced nephropathy. Fifth, we pointedly draw attention to the fact that there is some evidence to suggest publication bias, or at the very least funnel plot asymmetry. And finally, perhaps most importantly, we have explored the heterogeneity in results across studies in much greater detail than do Birck *et al*, and more directly address the relevance of this heterogeneity in the overall interpretation of study results. Two other meta-analyses have also recently been published, and similarly concluded that acetylcysteine is beneficial; however, these studies also failed to adequately address the issue of the considerable heterogeneity across studies [41,42]. Collectively, these three previously published meta-analyses unfortunately send a misleading bottom-line message to the medical community – that the evidence in favor of acetylcysteine is firm [43]. Many will correspondingly infer from these three 'positive' meta-analyses that there is no longer a need for primary research into the efficacy of acetylcysteine. Our global conclusion, meanwhile, is rather different, in that we more cautiously conclude that further data may be needed before a firm conclusion can be made regarding the efficacy of acetylcysteine. Two other very recent meta-analyses [44,45] make a similar conclusion to ours, though those meta-analyses do not include as many peer-reviewed and published studies as does our updated systematic review.

The presence of heterogeneity and/or publication bias can compromise the interpretation of meta-analyses and result in erroneous and potentially misleading conclusions [19,43]. A striking example of early meta-analysis producing misleading results is that of intravenous magnesium in the treatment of acute myocardial infarction. The results of two meta-analyses of several small clinical trials on this treatment suggested a reduction in arrhythmias and mortality [46,47]. Furthermore, an argument was made at the time for the use of magnesium therapy because of ease of use, favorable side effect profile and low cost [47,48]. However, the subsequent publication of ISIS-4, a large multi-center trial involving over 58,000 patients, showed not only the absence of significant reduction in arrhythmias or mortality with magnesium, but in fact a trend towards an increased risk of heart failure [49,50], results that have since been further validated by publication of the MAGIC trial [51]. The early meta-analyses on intravenous magnesium were perhaps influenced by publication bias and the combination of data from several small randomized controlled trials [52].

There are many parallels between the intravenous magnesium story and our meta-analysis findings for acetylcysteine. The marked heterogeneity of findings across studies, and the finding of funnel plot asymmetry (indicating possible publication bias), ought to be viewed as strong cautionary points against making firm conclusions about the efficacy of acetylcysteine. And while it is true that acetylcysteine is inexpensive, easy to use and has a favorable side-effect profile, it is probably premature to conclude scientifically that it is *definitely *efficacious based on data published to date. Our firm conclusion based on this meta-analysis of published trails is that although the data seem quite promising, the efficacy of acetylcysteine has not been definitively proven.

To isolate potential sources of heterogeneity we performed a meta-regression analysis exploring several clinical and study quality factors. There was no evidence of association between effect size and baseline serum creatinine, volume of contrast media, or diabetes mellitus, all independently identified risk factors for development of contrast-induced nephropathy [8,53]. However, whether the angiographic procedure was performed electively or as emergency showed a significant relation with the size of the acetylcysteine effect. The need to perform emergency cardiac angiography is common in patients presenting with suspected acute coronary syndromes. Patients undergoing emergency coronary angiography have been shown to have increased mortality and poor long-term survival, independent of the development of contrast-induced nephropathy [6,54].

Funnel plot asymmetry is often interpreted to indicate publication bias. However, it is important to consider that this asymmetry may also be due to other sources of bias that deserve further examination. In particular, fundamental disparities in study design, inconsistencies in methodological quality and differences in the definition of primary outcomes may have contributed to funnel plot asymmetry. Our meta-regression analysis explored the potential role of several study quality factors, and none were identified as statistically significant predictors of apparent acetylcysteine efficacy across trials. Nonetheless, it is quite possible that other unmeasured study quality factors may have contributed to biased results and accompanying funnel plot asymmetry.

Contrast-induced nephropathy continues to be an active subject matter for clinical investigation [55,56]. A definitive randomized clinical trial comparing fenoldopam, a selective type 1 dopamine receptor agonist, with placebo recently demonstrated no significant difference in the incidence of contrast-induced nephropathy or any secondary outcomes including 30 day mortality, need for dialysis, or re-hospitalization rates [56]. Another, recent randomized trial of 192 patients undergoing intravascular angiography compared prophylactic acetylcysteine with fenoldopam [57]. The results demonstrated a 9.6% absolute risk reduction in patients randomized to acetylcysteine (4.1% vs 13.7%, respectively). Although the authors conclude that acetylcysteine is superior to fenoldopam for prevention of contrast-induced nephropathy, there was notably no significant difference in serum creatinine at 48 h. Of interest, in subgroup analysis, the authors speculate that patients with low ejection fractions (<40%) may attain additional benefit with acetylcysteine.

## Conclusion

All of the above leads us to conclude that while acetylcysteine appears to be safe and inexpensive, its efficacy for the prevention of contrast-induced nephropathy remains unproven. The results of the trials that we reviewed to date should be viewed as early promising evidence of benefit, and suggest that it is now perhaps reasonable to use acetylcysteine in routine care because of its relative ease of use and safety. However, its true efficacy will remain uncertain unless a definitive well-designed multi-center trial is performed. Such a clinical trial will be most relevant if it addresses a priori clinically meaningful endpoints of renal insufficiency, rather than surrogate endpoints based on changes in creatinine levels alone, and further considers stratification on hypothesized important subgroups that may benefit such as those with a low ejection fraction [58].

## Competing interests

The authors declare that they have no competing interests.

## Authors' Contributions

SMB developed the study protocol, conducted literature search, screened articles for eligibility, extracted data, analyzed data, interpreted results, wrote and revised the manuscript. WAG contributed to protocol development, screened articles for eligibility, extracted data, analyzed data, interpreted results, and provided critique of successive drafts of the manuscript. Both authors read and approved the final manuscript.

## Pre-publication history

The pre-publication history for this paper can be accessed here:



## Figures and Tables

**Table 1:** Characteristics of studies reporting on the use of acetylcysteine for prevention of contrast-induced nephropathy.

**First Author**	**Patients**	**Diabetes**	**Elective Procedure**	**Primary Outcome**	Acetylcysteine Protocol*	**Hydration Protocol**	**Contrast Media**	Contrast Media Volume (mL)**	
								
								**NAC**	**Control**
**Allaqaband, 2002**^25^	85	41 (48%)	Unclear	> 44.2 μmol/L increase SCr at 48 hrs	600 mg PO bid pre/post	0.45%NS 1 mL/kg/hr 12 hr pre/post	Ioversol or Iodixanol	121.6	122
**Baker, 2003**^26^	80	34 (43%)	Unclear	> 25% increase SCr at 48 or 96 hrs	150 mg/kg IV 30 min pre & 50 mg/kg IV infusion 4 hrs post	Control 0.9%NS 1 mL/kg/hr 12 hr pre/post	Iodixanol	238	222
**Briguori, 2002**^27^	183	69 (38%)	Yes	> 25% increase SCr at 48 hrs or dialysis	600 mg PO bid pre/post	0.45%NS 1 mL/kg/hr 12 hr pre/post	Iopromide	194	200
**Diaz-Sandoval, 2002**^28^	54	21 (39%)	Yes	> 44.2 μmol/L or 25% SCr increase at 48 hrs	600 mg PO bid pre/post	0.45%NS 1 mL/kg/hr 2–12 hr pre & 12 hr post	Ioxilan	179	189
**Durham, 2002**^29^	79	38 (48%)	No	> 44.2 μmol/L increase SCr at 48 hrs	1200 mg PO 1 hr pre and 3 hr post	0.45%NS 1 mL/kg/hr 12 hr pre/post	Iohexol	77.4	84.7
**Efrati, 2003**^30^	49	26 (53%)	Yes	>25% increase SCr at 24 or 96 hrs	1000 mg PO bid day pre/post	0.45%NS 1 mL/kg/hr 12 hr pre/12 post	Iopromide	142	138
**Fung, 2004**^31^	91	48 (53%)	Yes	>44.2 μmol/L or 25% decrease in GFR	400 mg PO tid day prior/post	0.9%NS 100 ml/hr 12 hr pre/12 post	Iopromide	135.8	121
**Goldenberg, 2004**^32^	80	43 (54%)	No	>44.2 μmol/L	600 mg PO bid day prior/post	0.45%NS 1 mL/kg/hr 12 hr pre/12 post	Iopamidol	111	138
**Kay, 2003**^33^	200	75 (38%)	Yes	> 25% increase SCr at 48 hrs	600 mg PO bid × 4 (3 pre)	0.9%NS 1 mL/kg/hr 12 hr pre & 6 hr post	Iopamidol	130	120
**Kefer, 2003**^34^	104	13 (13%)	Unclear	> 44.2 μmol/L increase SCr at 24 hrs	1200 mg IV 12 hr pre & immediately post	D5W 20 mL/hr 12 hr pre & 24 hr post	Iopromide or Iohexol	NR	NR
**MacNeill, 2003**^35^	43	20 (46%)	Yes	> 25% increase SCr at 72 hrs	600 mg PO × 5 (2 pre)	Inpatient: 0.45%NS 1 mL/kg/hr 12 hr pre Outpatient: 0.45%NS 2 mL/kg/hr 4 hr pre & both 12 hr post	Iopromide or Ioxilan	103	116
**Oldemeyer, 2003**^36^	96	43 (45%)	Yes	> 44.2 μmol/L or 25% SCr increase at 48 hrs	1500 mg bid × 4 (1 pre)	0.45%NS 1 mL/kg/hr 12 hr pre/post	Iopamidol	134	127
**Shyu, 2002**^37^	121	77 (64%)	Yes	> 44.2 μmol/L increase SCr at 48 hrs	400 mg PO bid pre/post	0.45%NS 1 mL/kg/hr 12 hr pre/post	Iopamidol	119	115
**Vallero, 2002**^38^	100	23 (23%)	Unclear	> 44.2 μmol/L or 33% increase SCr at 48 hrs	600 mg PO bid pre/post	0.45%NS 1 mL/kg/hr 1–2 hr pre & 24 hr post	Iodixanol	187.8	219

Legend: NR = not recorded or available; NAC=acetycysteine; SCr=serum creatinine (for conversion to mg/dL divide by 88.4). *NAC administered with hydration protocol. **Data presented as means.

**Table 2:** Summary of quality indicators for studies of acetylcysteine for prevention of contrast-induced nephropathy.

**First Author**	**Jadad score**	**Inclusion/exclusion criteria specified**	**Randomization process described**	**Use of any blinding**	**Placebo-controlled**	**Reported loss to follow-up**	**Intention-to treat analysis**	**Potential important baseline differences**	**Power calculation**
**Allaqaband**^25^	3	yes/no	yes	yes	yes	no	no	no	yes
**Baker**^26^	2	yes/yes	yes	no	yes	no	yes	no	yes
**Briguori**^27^	1	yes/no	yes	no	no	no	unclear	yes	no
**Diaz-Sandoval**^28^	4	yes/yes	yes	yes	no	yes	unclear	yes	no
**Durham**^29^	5	yes/yes	yes	yes	yes	yes	unclear	no	yes
**Efrati**^30^	4	yes/yes	no	yes	yes	yes	unclear	no	no
**Fung**^31^	4	yes/yes	yes	no	no	no	yes	no	yes
**Goldenberg**^32^	5	yes/yes	yes	yes	yes	no	unclear	no	yes
**Kay**^33^	5	yes/yes	yes	yes	yes	yes	yes	no	yes
**Kefer**^34^	4	yes/yes	yes	yes	yes	yes	unclear	yes	no
**MacNeill**^35^	4	yes/yes	yes	no	yes	no	unclear	no	yes
**Oldemeyer**^36^	4	yes/yes	yes	yes	no	yes	unclear	no	no
**Shyu**^37^	3	yes/yes	yes	yes	no	yes	unclear	no	no
**Vallero**^38^	2	yes/yes	yes	no	no	no	unclear	yes	no

Legend: NR = not recorded or available; Jadad score range 0–5.

**Table 3:** Summary of outcomes of studies of acetylcysteine for prevention of contrast-induced nephropathy.

**First Author**	Contrast-induced nephropathy*		**Acetylcysteine Serum Creatinine (μmol/L)**		**Control Serum Creatinine (μmol/L)**		**Dialysis (N)**	
	
	**Acetylcysteine**	**Control**	**Baseline**	**Second SCr**	**Baseline**	**Second SCr**	**NAC**	**Control**
**Allaqaband**^25^	8/45 (18%)	6/40 (15%)	194.5	196.3	179.5	179.5	2	0
**Baker**^26^	2/41 (5%)	8/39 (21%)	163.6	156.5	154.7	159.1	0	0
**Briguori**^27^	6/92 (7%)	10/91 (11%)	134.4	130.8	136.1	135.3	0	1
**Diaz-Sandoval**^28^	2/25 (8%)	13/29 (45%)	146.7	135.5	137.9	166.2	0	0
**Durham**^29^	10/38 (26%)	9/41 (22%)	194.5	NR	203.3	NR	NR	NR
**Efrati**^30^	0/24 (0%)	2/25 (8%)	135.3	143.2	131.7	143.2	0	0
**Fung**^31^	8/46 (17%)	6/45 (13%)	200.7	216.6	209.5	212.2	NR	NR
**Goldenberg**^32^	4/41 (10%)	3/39 (8%)	176.8	176.8	168.0	165.3	0	0
**Kay**^33^	4/102 (4%)	12/98 (12%)	119.3	107.8	120.2	122.0	0	0
**Kefer**^34^	2/53 (8%)	3/51 (6%)	91.9	91.1	102.5	93.7	0	0
**MacNeill**^35^	1/21 (5%)	7/22 (32%)	167.1	168.0	168.0	210.4	NR	NR
**Oldemeyer**^36^	4/49 (8%)	3/47 (6%)	144.1	NR	146.7	NR	0	0
**Shyu**^37^	2/60 (3%)	15/61 (25%)	247.5	221.0	247.5	274.0	0	1
**Vallero**^38^	4/47 (9%)	4/53 (8%)	87.5	93.7	84	86.6	NR	NR

Legend: SCr = serum creatinine (for conversion to mg/dL divide by 88.4); NR = not recorded or available. *Values are numbers of patients with contrast-induced nephropathy/total number of patients in treatment group (%).
